# Plant actin depolymerizing factor: actin microfilament disassembly and more

**DOI:** 10.1007/s10265-016-0899-8

**Published:** 2017-01-02

**Authors:** Noriko Inada

**Affiliations:** The Graduate School of Biological Sciences, Nara Institute of Science and Technology, 8916-5 Takayama-cho, Ikoma-shi, Nara 630-0192 Japan

**Keywords:** Actin, Actin depolymerizing factor, Plant development, Plant–pathogen interactions, Stress response, Defense signaling

## Abstract

ACTIN DEPOLYMERIZING FACTOR (ADF) is a conserved protein among eukaryotes. The main function of ADF is the severing and depolymerizing filamentous actin (F-actin), thus regulating F-actin organization and dynamics and contributing to growth and development of the organisms. Mammalian genomes contain only a few *ADF* genes, whereas angiosperm plants have acquired an expanding number of *ADF*s, resulting in the differentiation of physiological functions. Recent studies have revealed functions of ADFs in plant growth and development, and various abiotic and biotic stress responses. In biotic stress responses, ADFs are involved in both susceptibility and resistance, depending on the pathogens. Furthermore, recent studies have highlighted a new role of ADF in the nucleus, possibly in the regulation of gene expression. In this review, I will summarize the current status of plant ADF research and discuss future research directions.

## Introduction

Actin, including both globular (G) and filamentous (F), regulates various cellular functions that are necessary for plants to grow and respond to environmental changes. Cytoplasmic streaming, which is regulated by F-actin and molecular mortor myosins, is an index of plant cell viability and is necessary in relatively large plant cells for efficient molecule transport (Tominaga and Ito 2015). Polarized growth of pollen tubes or root hair cells requires molecule delivery to the growing apex through F-actin. Regulation of the stomatal opening, which is important for plants to adapt to humidity changes, is dependent on F-actin (Higaki et al. 2010). F-actin also plays important roles in responses against microbial attacks (Day et al. 2011; Higaki et al. 2011; Takemoto and Hardham 2004).

Numerous actin-binding proteins regulate F-actin organization and dynamics, and thus various cellular and physiological functions (Henty-Ridilla et al. 2013; Higaki et al. 2007; Huang et al. 2015; Sun et al. 2013; van Gisbergen and Bezanilla 2013). Actin depolymerizing factor (ADF) is a relatively small (13–19 kDa) actin-binding protein that is conserved among eukaryotes. Historically, the first member of ADF was isolated from the porcine brain and named “cofilin,” indicating “cofilamentous protein” (Nishida et al. 1984). Proteins with F-actin depolymerizing activities, including cofilin, were independently identified in several organisms. Sequence analyses later revealed that those proteins were related (Moon and Drubin 1995). For this historical reason, ADF members in yeast and animal models are generally called ADF/cofilin. Although the major function of ADF/cofilin is recognized as severing and depolymerizing F-actin, it can also stabilize F-actin at higher concentration and promote nucleation of F-actin at very high concentration as shown by real-time imaging analysis of single F-actin (Andrianantoandro and Pollard 2006). In addition to the regulation of F-actin organization and dynamics, other cellular functions of ADF/cofilin have been proposed, such as inducing apoptosis by triggering the release of Ca^2+^ from mitochondria, and chaperoning G-actin to the nucleus, where G-actin functions in the regulation of gene expression and chromatin remodeling (Bernstein and Bamburg 2010).

In plants, the first ADF was isolated from lilies (*Lilium longiflorum*) in a genetic screen for clones that were preferentially expressed in anthers (Kim et al. 1993). Biochemical characterization of plant ADF was first performed with *Zea mays* ADF3 (ZmABP3 then renamed as ZmADF3), confirming its conserved activity to bind both F- and G-actin (Rozycka et al. 1995). It was also shown that *Arabidopsis thaliana* ADF1 (AtADF1) increases assembly of F-actin at higher concentration (Carlier et al. 1997). To date, ADF has been identified in many plants, including moss (*Physcomitrella patens*) (Augustine et al. 2008), rice (*Oryza sativa*) (Huang et al. 2012), and wheat (*Triticum aestivum*) (Ouellet et al. 2001). In planta, reduced expression of *AtADF1* increased bundling of F-actin, whereas overexpression of *AtADF1* reduced bundling (Dong et al. 2001b). Loss of *AtADF4* expression reduced severing frequency of F-actin (Henty et al. 2011).

Organisms in animal lineages possess only a few ADF/cofilin variants, whereas plants have acquired an expanded number of *ADF*s through evolution. For example, mammals such as mice (*Mus musculus*) and human (*Homo sapiens*) possess three ADF/cofilin members, whereas *A. thaliana* and *O. sativa* have 11 *ADF* loci (annotation in rice is referred to Huang et al. 2012), and *Populus trichocarpa* has 14 (Roy-Zokan et al. 2015). This expansion of gene number has allowed plant ADF to acquire functional specializations as suggested in many recent reports (e.g., Burgos-Rivera et al. 2008; Fu et al. 2014; Inada et al. 2016; Tang et al. 2015; Tian et al. 2009; Wang et al. 2009b).

Although the progress of animal ADF/cofilin research has been reviewed extensively (Bamburg and Bernstein 2010; Bamburg and Wiggan 2002; Bernstein and Bamburg 2010; Hild et al. 2014; Maciver and Hussey 2002; Van Troys et al. 2008), there have been no reviews focused on plant ADF to my knowledge. A review by Maciver and Hussey (2002) included research on plant ADFs, however, significant progress has been made since then. Therefore, in this review, I will summarize the current status of plant ADF research with much emphasis on the importance of ADF as a regulator of plant growth and stress responses. I will use “ADF/cofilin” to refer to animal proteins, and “ADF” for those in plants.

## Plant ADF variations

**Fig. 1:**
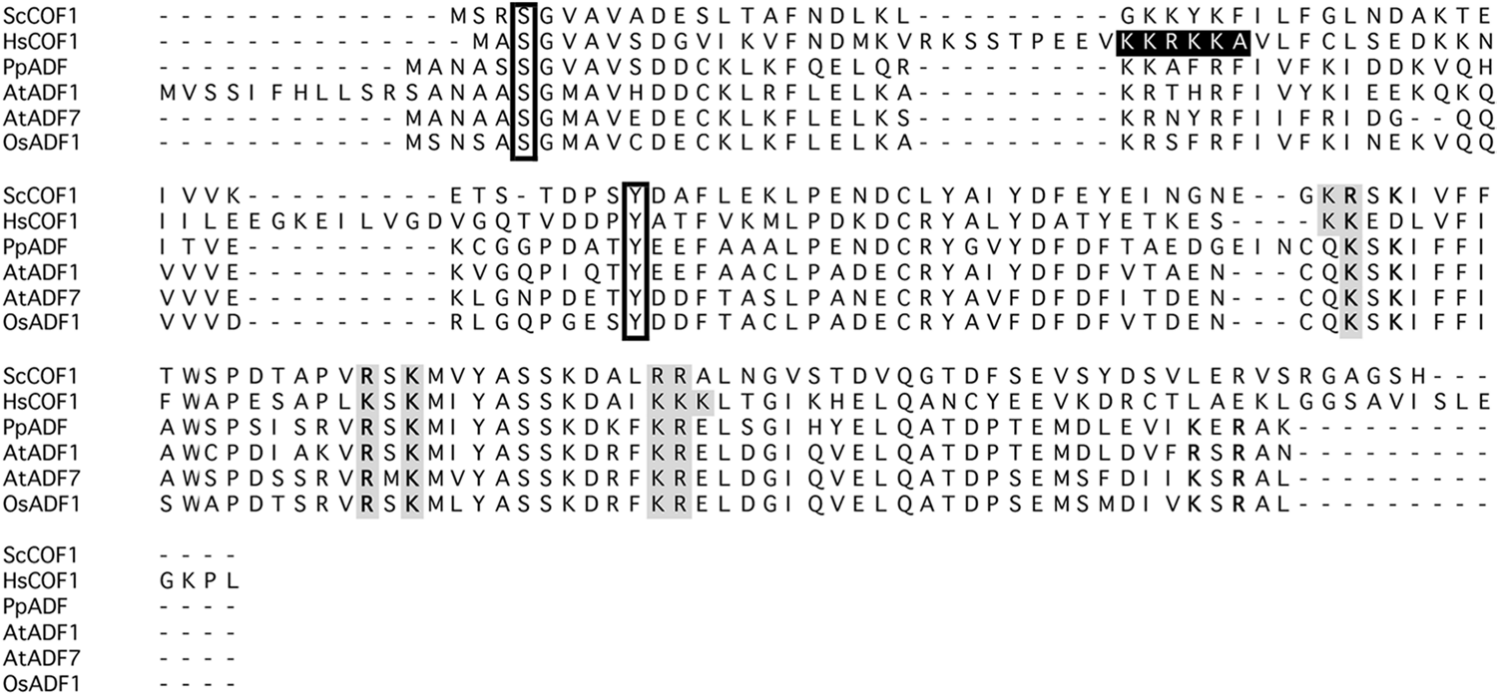
Sequence alignment of ADF. *Saccharomyces cereviseae* COF1 (ScCOF1), *Homo sapiens* COF1 (HsCOF1), *Physcomitrella patens* ADF (PpADF), *Arabidopsis thaliana* ADF1 and ADF7 (AtADF1 and AtADF7, respectively), and *Oryza sativa* ADF1 (OsADF1). *Boxes* indicate phosphorylation sites, and a *black box* indicates a nuclear localization sequence (NLS). Although angiosperm ADFs lack conventional NLS, their nuclear localization has been confirmed. *Shaded* residues indicate phosphatidylinositol 4,5-bisphosphate binding sites (Zhao et al. 2010), and residues indicated as *bold characters* are actin-binding sites (Dong et al. 2013)

As described above, angiosperms have acquired an expanded number of *ADF* genes, which is hypothesized to have derived from a single *ADF* in *P. patens* (Augustine et al. 2008; Roy-Zokan et al. 2015). These plant ADFs share protein structure with yeast and mammalian ADF/cofilin (Bowman et al. 2000, Fig. 1). Residues required for ADF activity and regulation, such as those for actin binding and phosphorylation (see below), have also been conserved in plant ADFs (Fig. 1).

The functional divergence and specialization resulting from this expansion of *ADF* genes are clearly manifested in differences in tissue- and organ-specific gene expression patterns. For example, among 11 members of *A. thaliana* *ADF*s, *AtADF1*, −*2*, −*3*, and −*4*, which are classified into subclass I, are expressed throughout the plants at relatively high levels, whereas *AtADF7* and *AtADF10*, which belong to subclass IIa, are expressed only in flowers, particularly in pollens. Another subclass of *ADF*s, *AtADF8* and *AtADF11* in subclass IIb, showed specific expression in root epidermal cells. Although both subclass III and subclass IV showed expression in a wide variety of tissues, the restricted expression of subclass III *AtADF5* and *AtADF9* in root apical meristem is not seen with subclass IV *AtADF6* (Ruzicka et al. 2007). The distinct expression patterns of *AtADF1* (subclass I), *AtADF5* (subclass III) and *AtADF6* (subclass IV) were also observed by Dong et al. (2001a), in which the restricted expression of *AtADF5* in root apical meristem was clearly noted. In rice, *OsADF2*, −*4*, and −*5* are expressed throughout the plant, whereas *OsADF9* and −*10* show specific expression in spikelets, and *OsADF1* and −*8* show high expressions in spikelets and are also expressed in other organs. *OsADF3* and −*11* are expressed throughout plants, except for roots. Promoter-GUS analysis also revealed the vascular specific expression of *OsADF1* and −*3* (Huang et al. 2012).

In addition to variations in expression patterns, biochemical activity also varies among ADFs. Particularly, it has been noted that pollen ADFs have structural and biochemical characters different than vegetative ADFs. The antibody raised against pollen-specific ADF did not cross-react with vegetative ADFs (Allwood et al. 2002; Smertenko et al. 2001). Pollen-specific LlADF1 exhibited much lower activity in actin depolymerization than maize ZmADF3, which is expressed in vegetative organs (Smertenko et al. 2001). Accordingly, pollen-specific AtADF7 is less efficient at disassembling F-actin than vegetative AtADF1 (Zheng et al. 2013). Although ADFs are commonly regulated by phosphorylation, as described later, pollen-specific LlADF1 seems to be exceptional (Allwood et al. 2002). Further differences have been seen in microscope images of the intracellular localization of pollen-specific ADFs. Vegetative ADFs exhibit nucleoplasmic localization in addition to co-localization with F-actin (Dong et al. 2013; Inada et al. 2016; Ruzicka et al. 2007; Tholl et al. 2011; Tian et al. 2009), whereas both immunostaining analysis and expression of fluorescent protein-tagged ADFs have shown the absence of pollen-specific ADFs in the nucleus (Allwood et al. 2002; Smertenko et al. 2001).

The differentiation of physiological function has also been reported for ADFs. Purified AtADF9 shows strong F-actin bundling activity, whereas AtADF1 exhibits classical ADF activity in F-actin destabilization (Tholl et al. 2011). The loss of *AtADF9* expression causes early flowering phenotypes, which are not complemented with the constitutive expression of distant *AtADF4* or *AtADF8* (Burgos-Rivera et al. 2008). Transient expression of *AtADF1*, −*5*, −*6*, −*7*, and −*10* (referred as *AtADF12* in the paper), but not that of *AtADF2*, −*3*, −*4*, and −*9*, inhibits the resistance of barley (*Hordeum vulgare*) against powdery mildew fungus *Blumeria graminis* f. sp. *hordei* (Miklis et al. 2007). Overexpression of *AtADF6*, but not that of *AtADF5*, inhibits the transport of RPW8.2-YFP, a resistant protein for powdery mildew fungi, to the membrane surrounding specialized infection hyphae, haustoria (Wang et al. 2009b). These differences in cellular function could be caused by differences in biochemical activity or regulatory mechanisms of ADFs, however, the underlying mechanisms remain unknown.

## Transcriptional regulation of *ADF*

**Fig. 2:**
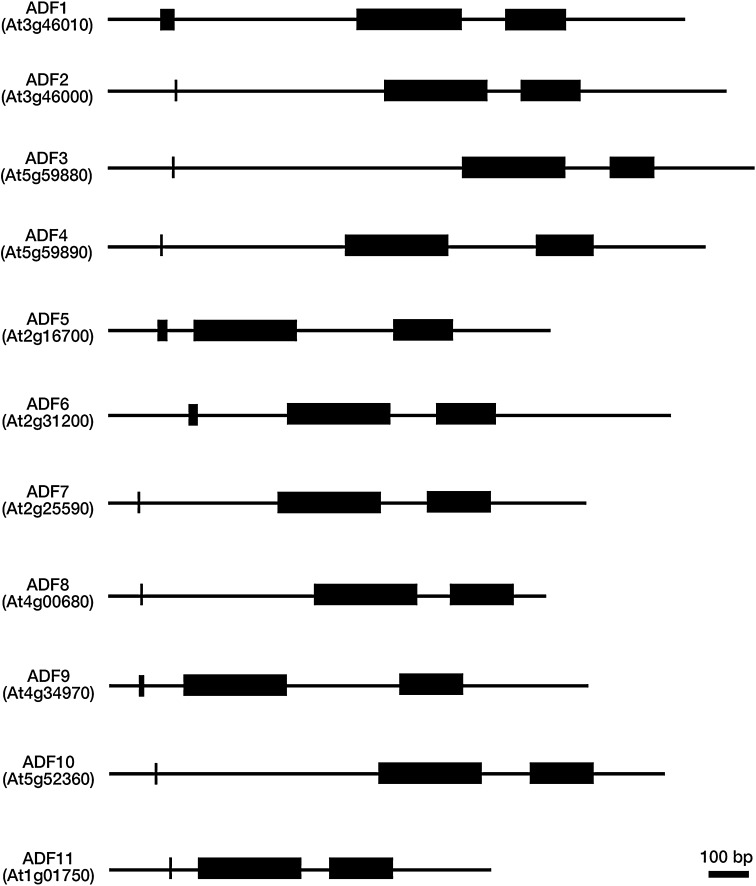
Gene structure of the *Arabidopsis thaliana* *ADF* family. *Black boxes* indicate exons. All 11 members of *AtADF*s comprise three exons, and the 1st exons contain either a single (only start codon) or a couple of amino acids. The 1st exon is followed by a long 1st intron in many *AtADF*s

Characterized angiosperm *ADF*s show well conserved gene structures, comprising three exons, including a very short 1st exon. As represented by the gene structure of *A. thaliana* 11 *ADF* members (Fig. 2), *ADF*s of angiosperms, including petunia (*Petunia hybrid*) (Mun et al. 2002) and rice (Huang et al. 2012), possess a very short 1st exon that contains only start codons, which is followed by a long 1st intron in most cases. Notably, mammalian ADF/cofilin sequences also have a short 1st exon (Maciver and Hussey 2002; Thirion et al. 2001), whereas *P. patens ADF* do not possess introns (Augustine et al. 2008).

The long 1st intron (1659 bp) of petunia *ADF* (*PhADF1*) functions in enhancing expression of *PhADF1* (Mun et al. 2002). When the *GUS* gene was fused to the 1.7 kb upstream region of *PhADF1* and expressed in *A. thaliana*, GUS expression was only seen in the vascular area, whereas a construct containing *PhADF1* 1.7 kb promoter-1st intron-*GUS* showed strong GUS activity throughout the plant. Notably, when the *PhADF1* genomic region from the 1.7-kb native promoter to the 2nd intron was fused upstream of *GUS*, the transgenic plant showed much lower expression levels than plants expressing *PhADF1* promoter-1st intron-*GUS*, suggesting that *PhADF1* introns function differently in the regulation of gene expression (Mun et al. 2002). The enhancement of gene expression by the 1st intron is conserved in another member of petunia *ADF, PhADF2*, as well as in *AtADF1* (Jeong et al. 2007). In addition, inserting the *PhADF1* 1st intron can alter both expression level and pattern of other genes. *A. thaliana* profilin 5 (*PRF5*) normally expresses only in reproductive organs. Insertion of the *PhADF1* 1st intron between the 1st exon of *PRF5* and *GUS* gene results in *PRF*5 expression throughout the plant. Owing to the high A/T composition, no meaningful conserved sequences were found in these *ADF* 1st intron sequences (Jeong et al. 2007); thus, the regulatory mechanisms remain unknown.

The structure of the promoter region has been examined in petunia and rice. For petunia *PhADF1*, several potential transcription factor-binding sites and hormone response domains were found in the upstream region of the gene body (Mun et al. 2002). Huang et al. (2012) performed comprehensive analyses on the promoter structure and expression pattern of all the 11 rice *ADF*s and found the presence of stress-related *cis*-acting elements, including ABA-responsive element, dehydration-responsive element/C-repeat, and low-temperature responsive element in the 1-kb promoter regions. Accordingly, the expression of some *OsADF*s is induced by stresses such as low temperature, drought, ABA, and salt (Huang et al. 2012).

The alternative splicing of ADF/cofilin that produces variations in both the ADF/cofilin sequence and function has been reported in human (Thirion et al. 2001) and *Caenorhabditis elegans* (McKim et al. 1994; Ono and Benian 1998; Ono et al. 2008). The *A. thaliana* database (TAIR, http://www.arabidopsis.org/) indicated that *AtADF1*, −*3*, −*4*, and −*5* have splicing variants. For *AtADF1* (At3g46010), which has a 139 amino acid long At3g46010.1 variant and 150 amino acid long At3g46010.2 variant, the sequence of At3g46010.1 has been identified in the *A. thaliana* EST database (Dong et al. 2001a), and its biochemical activity has been analyzed (Dong and Hong 2013; Dong et al. 2013). However, At3g46010.2, which has an additional 11 residues following the start codon, has not been characterized and it is not known whether the two variants differ in expression pattern or activity in F-actin depolymerization.

## Regulation of ADF activity

The activity of ADF/cofilin in animal models is post-transcriptionally regulated by many factors, including environmental pH and phosphorylation. Interactions with 14-3-3 protein, phosphatidylinositol 4,5-bisphosphate (PIP_2_), and actin-interacting protein (AIP) also affect the activity of ADF/cofilin (Bernstein and Bamburg 2010).

These regulatory factors are well conserved in plants. Regarding the pH dependency of ADF activity, plant ADFs, such as those in wheat (TaADF) (Ouellet et al. 2001), tobacco (*Nicotiana tabacum*, NtADF1) (Chen et al. 2002), lily (LlADF1), and maize (ZmADF3) (Allwood et al. 2002), show high activity in binding to F-actin at pH 6.5, whereas they bind to G-actin more efficiently at pH 8.0, similarly to ADF/cofilin in animal models. This pH-dependent change in ADF/cofilin activity is possibly caused by the pH-dependent conformational changes of actin (Blondin et al. 2002).

Other factors known to regulate activities of plant ADFs are summarized below.

## Phosphorylation

Phosphorylation of serine (Ser) at the N-terminus is a conserved regulatory mechanism of ADF activity in F-actin disassembly. Upon phosphorylation, ADF becomes inactivated and loses its F-actin-binding ability (Blanchoin et al. 2000; Ressad et al. 1998). In animals, this serine is located at the 3rd amino acid of ADF/cofilin, whereas many plant ADFs have the 6th serine (S6) phosphorylated (Fig. 1).

This S6 phosphorylation of plant ADFs was first shown with ZmADF3. A phosphomimetic mutation at S6 made ZmADF3 lose its ability to bind F- or G-actin (Smertenko et al. 1998). Microscopic observation also confirmed the loss of the F-actin-binding ability of plant ADFs by phosphorylation. Porter et al. (2012) produced *A. thaliana* plants stably overexpressing a coding sequence of *AtADF4* conjugated with green fluorescent protein (GFP) and found that the co-localization of AtADF4-GFP with F-actin was disrupted when a phosphomimetic mutation was incorporated at S6 of AtADF4 (Porter et al. 2012). Similarly, phosphomimetic AtADF1 that was conjugated to GFP and transiently expressed in onion peels lost its filamentous localization pattern (Dong and Hong 2013). However, this reduction in co-localization with F-actin by the incorporation of a phosphomimetic mutation was not reproduced in another study that used transgenic plants stably expressing the genomic region of *AtADF4* containing a 1.5-kb native promoter, exons, introns, and 3′ UTR (Inada et al. 2016). Biochemical analysis needs to be performed using those plants expressing the mutant form of ADFs to conclusively determine the relationship between the phosphostatus of ADF and its intracellular localization.

In animal systems, both kinases and phosphatases regulating the phosphostatus of ADF/cofilin were identified and well characterized (Bernstein and Bamburg 2010; Mizuno 2013). In plants, several candidate ADF kinases have been reported. Calcium-dependent protein kinase (CDPK) possibly phosphorylates ZmADF3 (Smertenko et al. 2001) and was partially purified (Allwood et al. 2001). However, chelating Ca^2+^ by EGTA does not abolish ZmADF3 phosphorylation, thus other factor(s) can also function in ZmADF3 phosphorylation (Smertenko et al. 1998). An in vitro assay using purified proteins showed that AtCDPK6 phosphorylates AtADF1 (At3g46010.1) at S6. Induced expression of *AtCDPK6* increased F-actin bundling *in planta*, a phenotype similar to that of *Atadf1*, indicating the function of AtCDPK6 in the suppression of ADF activity (Dong and Hong 2013). Meanwhile, a 52-kD kinase that requires Mn^2+^ for its activity, which is a property of plant receptor-like protein kinases, was identified to phosphorylate wheat TaADF (Ouellet et al. 2001). Recently, *A. thaliana* CASEIN KINASE1-LIKE PROTEIN2 (CKL2) was identified to regulate activities of subclass I AtADFs (AtADF1, −2, −3, and −4) and stomatal closure upon ABA treatment or drought (Zhao et al. 2016). Knockout mutant *ckl2* is unable to close stomata under ABA treatment or drought stress, and this phenotype is partially suppressed by *Atadf4*. CKL2 physically interacts with and phosphorylates subclass I AtADFs. It will be of interest to determine whether CKL2 regulates the functions of AtADF in other pathways, such as pathogen responses (Inada et al. 2016; Porter et al. 2012, see below).

Although phosphorylation is an important regulatory mechanism of ADF activity in F-actin disassembly, its importance in ADF function may vary depending on the variants or physiological events that ADF is involved in. In *P. patens*, which has a single *ADF* in the genome, phosphoregulation of ADF is essential for cell growth, because both phosphodeficient and phosphomimetic mutants of *PpADF* expression only partially complement the effect caused by the loss of *PpADF* expression (Augustine et al. 2008). Similarly, neither phosphodeficient nor phosphomimetic mutants of AtADF4 complement the enhanced resistance against powdery mildew disease in *Atadf4* (Inada et al. 2016, see below). On the other hand, the phosphomimetic mutant of AtADF4 did complement the *Atadf4* susceptible phenotype against bacterial pathogens (Porter et al. 2012). Furthermore, the F-actin-binding activity of ZmADF3 was inhibited by the incorporation of a phosphomimetic mutation, whereas its activity on F-actin disassembly was not (Smertenko et al. 1998).

In addition to S6, tyrosine 68 (T68) is also a residue that is commonly phosphorylated in mammalian, chicken (*Gallus gallus*), and *Xenopus* cofilin, but is not present in mammalian or chicken ADF (Bernstein and Bamburg 2010). Upon phosphorylation of T68, ubiquitination and degradation of cofilin is increased; therefore regulating the level of cofilin protein (Yoo et al. 2010). This tyrosine residue is conserved in some plant ADFs (Fig. 1), although there have been no reports to date showing the phosphorylation of ADF tyrosine residue or ubiquitination of plant ADFs.

## Phosphatidylinositol 4,5-bisphosphate

Phosphatidylinositol 4,5-bisphosphate (PIP_2_) is an established regulator of mammalian ADF/cofilin, and upon the binding of PIP_2_, the ADF/cofilin activity is inhibited (Bernstein and Bamburg 2010). The only example of PIP_2_ regulation on plant ADF is shown with ZmADF3. Biochemical analysis showed that the activity of recombinant ZmADF3 on F-actin depolymerization was inhibited by both PIP_2_ and phosphatidylinositol 4-monophosphate (PIP) (Gungabissoon et al. 1998).

ADF/cofilin interacts with PIP_2_ through a large, positively charged surface, consisting of lysine (K) and arginine (R) (Zhao et al. 2010). These K–R clusters are conserved in plant ADFs (Fig. 1), indicating the conservation of the PIP_2_ regulation of plant ADFs.

## 14-3-3 proteins

14-3-3 proteins, which were named after their particular elution and migration patterns on two-dimensional DEAE cellulose chromatography and starch gel electrophoresis, are present in all eukaryotic organisms including plants. 14-3-3 proteins act as phosphosensors, monitoring the phosphostatus of a specific site, and translating the information into a change in the activity of the target protein. In plants, 14-3-3 proteins are involved in various aspects of plant physiology including biotic responses (Lozano-Duran and Robatzek 2015). In mammals, 14-3-3ζ binds and stabilizes phosphorylated ADF/cofilin, thus negatively regulating the ADF/cofilin activity of severing and depolymerizing F-actin (Gohla and Bokoch 2002).

*Arabidopsis thaliana* 14-3-3λ interacts with AtADF1 and regulates F-actin organization in vivo (Zhao et al. 2015). However, contrary to what is expected from animal 14-3-3 that stabilizes phosphorylated ADF/cofilin thus suppresses F-actin depolymerizing activity of ADF/cofilin, a knockout mutant of *14-3-3λ* exhibited an elongated hypocotyl phenotype, similarly to *Atadf1* and *Atadf4*. Authors also observed that a *14-3-3λ* loss-of-mutant increased the phosphorylation level of AtADF1 thus hindered the proper localization of AtADF1 to F-actin (Zhao et al. 2015). Therefore, the regulatory mechanism of ADF by 14-3-3 may differ between animals and plants. As ADF phosphorylation is an important component of the regulation by 14-3-3, it is necessary to understand the entire mechanism, in which both kinase and 14-3-3 are involved, to reveal the regulatory mechanisms of ADF function.

## Actin-interacting protein

Actin-interacting protein (AIP) is a conserved WD-repeat protein, which is required for efficient disassembly of F-actin by ADF/cofilin (Ono et al. 2004). Recently, it was demonstrated that AIP1 severs F-actin that is fully decorated with ADF/cofilin, possibly by competing with ADF/cofilin in F-actin binding, thus lowering the concentration of ADF/cofilin, which promotes F-actin severing by ADF/cofilin (Chen et al. 2015; Gressin et al. 2015).

Several papers have described a conserved role of plant AIP in promoting ADF-mediated F-actin disassembly. Allwood et al. (2002) identified interaction between *A. thaliana* AIP1 and lily LlADF1 using the yeast two hybrid assay and co-immunostaining analysis. They also performed an in vitro assay and demonstrated that *A. thaliana* AIP1 promotes F-actin depolymerization by LlADF1 (Allwood et al. 2002). Two *AIP1* genes are encoded in the *A. thaliana* genome, and suppression of *AIP1* expression impairs the growth and development of plants and induces increased bundling of F-actin, which is a phenotype seen in *adf* mutants (Ketelaar et al. 2004). Overexpression of *AtAIP1* reduces F-actin bundling and induces swollen root hairs (Ketelaar et al. 2007). Knockout of *P. patens AIP1*, which is encoded by a single gene in the genome, causes severe defects in the tip growth, which is partially rescued by *PpADF* expression. F-actin in the *P. patens aip1* mutant was more static and slower in remodeling than that in wild type moss, thus providing support that moss AIP functions in F-actin regulation through ADF (Augustine et al. 2011).

Shi et al. (2013) showed that rice AIP1 (OsAIP1) could promote ADF activity in severing and depolymerizing F-actin by an in vitro assay. In vivo, a knockdown of *OsAIP1* promotes, whereas its overexpression inhibits, F-actin assembly. Co-sedimentation analysis showed the enhancement of F-actin disassembly by OsAIP1 only in the presence of ADF. The increase in frequency of in vivo F-actin severing by OsAIP1 in the presence of ADF was also confirmed by observation with total internal reflection fluorescence microscopy (TIRFM), by which the organization and movement of F-actin at the cell surface can be visualized at high resolution, thus confirming the role of OsAIP in regulating F-actin dynamics (Shi et al. 2013).

## Function of ADF in plant development and growth

ADF controls cell elongation via the regulation of F-actin organization, thus contributes to plant growth. The knockdown of *PpADF* suppresses the tip growth of moss (Augustine et al. 2008), whereas the reduction of single *ADF* expression in angiosperms often exhibits cell elongation. Cotton (*Gossypium hirsutum*) *ADF1* (*GhADF1*) expresses abundantly in fiber cells, and *GhADF1* downregulation alters the cell wall morphology and elongates length of fiber cells (Wang et al. 2009a). Knockdown of *AtADF1* significantly elongates organ length, such as that of cotyledons, hypocotyls, roots, and stems, whereas over-expression significantly shortens their lengths (Dong et al. 2001b). The effect of *AtADF1* overexpression on the elongation of dark-grown hypocotyl is suppressed by the introduction of mutations in the actin-binding domain of AtADF1 (Dong et al. 2013). The loss of *AtADF4* expression also causes hypocotyl elongation when grown in the dark (Henty et al. 2011), but shortens the root hairs in the standard growth condition with light (Inada et al. 2016). Therefore, ADF regulates cell elongation differently depending on tissues.

Organ morphology is also affected by altered *ADF* expression. *AtADF1* overexpression causes wavy hypocotyls (Dong et al. 2001b), and *Atadf4* exhibits wavy and flattened mature leaves, which is not observed in *Atadf1* or *Atadf3* (Inada et al. 2016). However, as this phenotype in leaf shape was more strongly manifested in plants in which expressions of all of subclass I *AtADF*s were suppressed, other members of subclass I AtADFs, AtADF1, −2, and −3, possibly contribute to the regulation of leaf shape redundantly (Inada et al. 2016). The alteration in leaf shape is also reported with induced *AtADF2* knockdown plants (Clement et al. 2009). Ethanol-inducible *AtADF2* RNAi plants exhibited more serration of leaves at the circumference. In addition, ectopic appearance of lateral roots, and small, shrunken, and bent siliques that contained less fertile seeds were observed (Clement et al. 2009). Although leaf serration and phenotypes of lateral root and silique were not observed in subclass I *AtADF*s knockdown lines (Inada et al. 2016), this difference in phenotypes could be due to difference in the level of *AtADF2* expression suppression.

The role of ADF in regulating flowering timing has been indicated in two reports. The downregulation of *AtADF1* expression significantly delayed the timing of flowering, whereas plants overexpressing *AtADF1* flowered with timing similar to that of the wild type (Dong et al. 2001b). *AtADF9* expresses at relatively low levels in seedlings and mature tissues, but at very high level in the callus (Ruzicka et al. 2007). The knockout mutant *Atadf9* shows delayed growth and early flowering (Burgos-Rivera et al. 2008). This early flowering phenotype is correlated with a lower expression of *FLC* and higher expression of other flowering-related genes such as *CO* and *LFY* in *Atadf9* (Burgos-Rivera et al. 2008). As mentioned above, this early flowering phenotype of *Atadf9* was not complemented with *AtADF4* or *AtADF8* expression, which is correlated with the results that *Atadf4* showed no changes in flowering timing (Inada et al. 2016). Thus, AtADFs functionally differentiate regarding the regulation of flowering timing.

## Function of ADF in plant responses to abiotic and biotic stresses

The acquisition of multiple ADF genes in angiosperm lineages has made it possible for plant ADFs to obtain a role in responses to both abiotic and biotic stresses, as emphasized in recent studies.

## Function of ADF in abiotic stresses

Many papers reported increased protein accumulation or gene expression of ADF, particularly ADFs of grasses, when plants are subjected to various abiotic stresses.

Drought stress induced accumulation of OsADF in leaves of rice seedlings (cv. CT9993 and IR62266) (Salekdeh et al. 2002) and accumulation of *OsADF2* expression in the rice variety Azucena (Yang et al. 2003). OsADF3 accumulates in the sheath of the cultivars Nipponbare and Zhonghua 8 during drought and osmotic stresses, but not during cold or salt stresses (Ali and Komatsu 2006; Huang et al. 2012). On the contrary, in roots, salt stress induces OsADF3 accumulation in Nipponbare (Huang et al. 2012; Yan et al. 2005), indicating a tissue-specific function of OsADF3. In a cDNA library of smooth cordgrass (*Spartina alterniflora*), which is a halophyte that is capable of tolerating salinity as high as double that of sea water, the gene expression of *ADF* is induced when plants are subjected to salinity stress (Baisakh et al. 2008).

ADF could also be involved in the tolerance to both high- and low-temperature stresses. Smooth cordgrass *ADF* was induced under heat stress (Baisakh and Subudhi 2009). The expression level of *OsADF3* in anthers is higher in heat-tolerant cultivars than less tolerant ones (Gonzalez-Schain et al. 2016). However, wheat *ADF* (*TaADF*) has been identified as a gene of which expression is rapidly and strongly upregulated during low temperatures (Danyluk et al. 1996). TaADF accumulates at higher levels when wheat plants are subjected to low temperatures. The accumulation of TaADF is much higher in freezing-tolerant cultivars than less tolerant ones, thus suggesting its role in cold acclimation (Ouellet et al. 2001).

The exogenous application of the plant hormone, abscisic acid (ABA), induces swelling, root hair formation, and initiation of lateral root primordia in tips of young seminal rice roots. Proteomic analysis revealed that two ADFs of rice (Taichung native 1), called as ADF-1 and -2 in the paper, accumulate in response to ABA application. These OsADFs exhibit the same amino acid sequences; thus, are possibly the same protein of phosphorylated or dephosphorylated form (Chen et al. 2006). This ABA-induced OsADF was later identified as OsADF3 (Huang et al. 2012). ABA, but not salicylic acid, jasmonic acid, and ethylene, induces *TaADF3* expression. The induction of *TaADF3* expression was also seen during drought and cold stresses, but not salt stress or wounding (Tang et al. 2015).

The only example for the functional indication of ADFs during abiotic stresses is from a study of OsADF3 overexpressed in *A. thaliana. OsADF3* overexpressing *A. thaliana* plants became more tolerant against both drought and osmotic stresses (Huang et al. 2012). Further genetic studies are necessary to clarify the role of ADF in abiotic stresses in the future.

## Function of ADF in biotic stresses

The role of plant ADFs in interactions with pathogenic microbes, including nematodes, bacteria, viruses, and fungi, in both compatible (results in pathogen growth and proliferation) and incompatible (results in arrest of pathogen growth and often in plant cell death) interactions, has been recently highlighted in many reports. Generally, ADF positively regulates plant susceptibility in compatible interactions and plant resistance in incompatible interactions, although there are exceptions.

In a compatible interaction with nematodes, *A. thaliana* ADF2 acts as a positive regulator to establish the interaction, i.e., helps the nematode infecting the host plant. The root knot nematode *Meloidogyne incognita* forms giant cells in infected host roots, where the dynamic reorganization of host F-actin is observed (de Almeida Engler et al. 2004). Clement et al. (2009) found that *AtADF2* gene expression is increased in cells containing giant cells. Transgenic plants with reduced *AtADF2* expression exhibit severely delayed maturation of giant cells and significantly reduced egg production, which are correlated with increased bundling of F-actin in giant cells in *AtADF2* knockdown plants (Clement et al. 2009).

In an incompatible interaction with the bacterial pathogen, *Pseudomonas syringae* pv. *tomato* (*Pst*) DC3000 harboring the avirulent protein AvrPphB, *A. thaliana* ADF4 plays a role in host resistance. The knockout mutant of *AtADF4* shows increased susceptibility against *Pst* DC3000 AvrPphB, but not against lines carrying AvrRps2 or AvrB (Tian et al. 2009). This increased susceptibility is correlated with reduced *RPS5* expression, a gene responsible for resistance against *Pst* DC3000 AvrPphB (Porter et al. 2012).

Soybean mosaic virus (SMV) establishes a compatible interaction with the soybean (*Glycine max*), and SMV P3, a protein with unknown function, interacts with soybean ADF2. This P3-GmADF2 interaction was shown in a yeast two hybrid assay and bimolecular fluorescent complementation assay. As SMV P3 possibly functions in virus replication, systemic infection, pathogenicity, overcoming resistance, and cell-to-cell movement, the authors suggested that the interaction between P3 and GmADF2 could facilitate the cell-to-cell movement of P3 (Lu et al. 2015).

The functions of ADFs in both compatible and incompatible interactions with fungi are highlighted in many reports. In an incompatible interaction with stem rust *Puccinia graminis* f. sp. *tritici* race QCCJ, barley *rpg4*-mediated resistance locus 1 (RMRL1) mediates a resistance against this fungus. This locus contains *HvRga1, Rpg5*, and *HvAdf3*, although suppression of *HvAdf3* expression alone did not induce resistance against *P. graminis* (Wang et al. 2013). In an incompatible interaction with *P. striiformis* f. sp. *tritici* CYR23, the *ADF7* expression of the wheat genotype Suwon 11 (*TaADF7*) was sharply elevated, whereas an infection by compatible CYR31 caused much smaller changes in *TaADF7* expression. Reduced expression of *TaADF7* caused increased susceptibility to CYR23 (Fu et al. 2014). On the other hand, *TaADF3* functions in an opposite manner in interactions with *P. striiformis* f. sp. *tritici*. The expression of *TaADF3* is downregulated by incompatible CYR23, but upregulated by compatible CYR31. The suppression of *TaADF3* expression enhances resistance to both incompatible CYR23 and compatible CYR31, and impedes both fungal entry and haustoria formation of CYR31 (Tang et al. 2015).

In addition, we recently reported a function of *A. thaliana* subclass I ADFs, particularly AtADF4, in a contribution to the susceptibility against compatible powdery mildew fungus, *Golovinomyces orontii* (Inada et al. 2016). We found that the knockout mutant of *Atadf4* shows significantly enhanced resistance against *G. orontii*. The altered susceptibility is not observed in *Atadf1*, whereas *Atadf3* shows enhanced yellowing, which is a sign of enhanced immune response, at later infection stages. Transgenic plants in which the expression of all of four subclass I *ADF*s is suppressed (*ADF1-4Ri*) exhibit even more enhanced resistance against *G. orontii* and no visually noticeable mycelium formation at 2 weeks post inoculation, indicating the functional redundancy among subclass I ADFs in response to *G. orontii*. This increased resistance against *G. orontii* in *Atadf4* and *ADF1-4Ri* is correlated with the infected cell-specific accumulation of reactive oxygen species and cell death, which are mediated by both salicylic acid and jasmonic acid. From this prominently enhanced resistance of *Atadf4* and *ADF1-4Ri*, significant reorganization of F-actin in those mutants and transgenic plants was expected. However, we observed no changes in F-actin organization in uninfected *Atadf4* and *ADF1-4Ri*, and only a transient increase in F-actin density in infected *ADF1-4Ri* cells at a very early infection stage. These unexpected results prompted us to examine another function of ADF in the nucleus. An expression of a construct containing an *AtADF4* genomic sequence fused to GFP and a nuclear exporting signal revealed the importance of nuclear localization of AtADF4 in the *A. thaliana*–*G. orontii* interaction, suggesting an unconventional role of AtADF4 (Inada et al. 2016, further discussed below).

## Perspective

As summarized above, ADF is involved in many aspects of plant physiology. Particularly, as plant ADFs can function in resistance to various abiotic and biotic stresses, the achievements obtained from plant ADF research could be translated into applications, such as a production of stress-tolerant plants.

For future plant ADF research, I would like to propose two major issues that need to be resolved.

1. Elucidation of regulatory mechanism of plant ADF: although the cellular function of plant ADF in regulating F-actin organization and dynamics has been well characterized by biochemical analyses and observation of in vivo F-actin organization or dynamics in the presence or absence of ADF, the regulatory mechanism of plant ADF activity remains elusive. Recently, CKL2 was identified as a kinase of *A. thaliana* subclass I ADF in regulating stomatal opening (Zhao et al. 2016); however, it remains unknown whether the same kinase regulates the other functions of ADF, such as those in pathogen responses. Plant ADF phosphatases have not been identified. In addition, there are only a few examples regarding regulation of plant ADF by 14-3-3, PIP_2_ or AIP.
  ADF regulatory factors can possibly regulate event-specific function of ADF, thus could be a target of modification to produce stress tolerant plants. Therefore, more efforts should be paid to understand the plant ADF regulatory pathway in the future. Forward genetic analysis to isolate mutants that either revert or aggravate *adf* phenotypes as well as proteomic analyses of ADF-interacting partners will contribute to the identification of the ADF regulatory factors thus clarify the regulatory mechanism of ADF.
2. Understanding the mechanism of gene expression regulation by ADF: in addition to the conventional role of ADF in the destabilization of F-actin at the cell surface, the role of plant ADF in regulating gene expression has been repeatedly suggested. In a knockout mutant of *AtADF9*, which shows a reduction in plant size and early flowering under long day conditions, the expression level of genes related to flowering, such as *FLOWERING LOCUS C*, is altered (Burgos-Rivera et al. 2008); the increased susceptibility of *Atadf4* against the bacterial pathogen *Pst* DC3000 AvrPphB is correlated with a significant reduction in expressions of *RPS5*, a resistance gene required for the recognition of AvrPphB, and *FRK1*, a marker of innate immunity, in response to *Pst* DC3000 AvrPphB infection (Porter et al. 2012); in plant–powdery mildew fungus interaction, *ADF1-4Ri* exhibited significantly higher levels of *PR1* expression, even in uninfected leaves (Inada et al. 2016). In the case of plant–powdery mildew fungus interactions, the importance of nuclear localization of ADF has also been shown. Furthermore, in papers describing the role of ADF in plant–pathogen interactions, a correlation between changes in F-actin organization and plant phenotypes caused by ADF loss is often not shown or is unclear. For example, an arrangement of F-actin in mesophyll cells, the main site of attack by *P. striiformis* f. sp. *tritici*, was not altered in plants with reduced *TaADF3* expression and enhanced resistance against this fungus (Tang et al. 2015). Therefore, this unconventional role of ADF could play an important role in regulating a wider range of plant physiology.
  There have been a couple of implications for the mechanism of ADF/cofilin in regulation of transcription and gene expression. Nishida et al. reported that DMSO or heat shock treatment induces cofilin-dependent formation of nuclear actin rods and speculated that cofilin regulates the amount of available G-actin through the formation of actin rods (Nishida et al. 1987). As G-actin is a component of RNA polymerase and nuclear remodeling complex and thus functions in regulating transcription and gene expression (Zheng et al. 2009), ADF/cofilin could contribute to the regulation of transcription and gene expression through the formation of actin rods. The formation of nuclear actin rods that contain ZmADF3 is also observed in maize roots treated with cytochalasin (Jiang et al. 1997). More recently, mouse cofilin-1 was shown to form a complex with actin and RNA polymerase II (pol II) and to play a key role in pol II transcription (Obrdlik and Percipalle 2011), although the functional form of pol II-associated actin is not known. In addition to G-actin, nuclear F-actin has also emerged as a regulator of various nuclear events including transcription and gene expression (Belin and Mullins 2013). ADF/cofilin could affect transcription and gene expression through its function in regulation of the nuclear F-actin structure, although the mechanism that nuclear F-actin regulates transcription and gene expression is still unclear.
  To elucidate the nuclear function of ADF, multifaceted approach including imaging analysis of dynamic nuclear structures such as nuclear F-actin (Baarlink et al. 2013) and actin rods, biochemical analysis to identify interaction partners of nuclear ADF followed by genetic analysis of the function of identified proteins, detailed examination how altered *ADF* expression affect gene expression and chromatin structure should be performed.

In summary, further studies on plant ADFs will not only contribute to understanding of plant physiology, but also greatly contribute to basic cell biology.
