# Correction to: Plant actin depolymerizing factor: actin microfilament disassembly and more

**DOI:** 10.1007/s10265-018-1013-1

**Published:** 2018-02-21

**Authors:** Noriko Inada

**Affiliations:** The Graduate School of Biological Sciences, Nara Institute of Science and Technology, 8916-5 Takayama-cho, Ikoma-shi, Nara 630-0192 Japan

## Correction to: J Plant Res (2017) 130:227–238 10.1007/s10265-016-0899-8

The article “Plant actin depolymerizing factor: actin microfilament disassembly and more”, written by “Noriko Inada”, was originally published Online First without open access. After publication in volume 130, issue 2, page 227–238 the Botanical Society of Japan decided to opt for Open Choice and to make the article an open access publication. Therefore, the copyright of the article has been changed to © The Author(s) 2018 and the article is forthwith distributed under the terms of the Creative Commons Attribution 4.0 International License (http://creativecommons.org/licenses/by/4.0/), which permits use, duplication, adaptation, distribution and reproduction in any medium or format, as long as you give appropriate credit to the original author(s) and the source, provide a link to the Creative Commons license, and indicate if changes were made.

